# Not just species recording: the potential of citizen science for habitat monitoring

**DOI:** 10.1007/s10980-025-02155-4

**Published:** 2025-08-26

**Authors:** Ailidh E. Barnes, Michael J. O. Pocock, Maddie M. Harris, Niki Newton, Robert A. Robinson

**Affiliations:** 1 British Trust for Ornithology, The Nunnery, Thetford, Norfolk, IP24 2PU USA; 2 UK Centre for Ecology and Hydrology, Crowmarsh Gifford, Wallingford, Oxfordshire OX10 8BB UK; 3 Joint Nature Conservation Committee, Quay House, 2 East Station Road, Fletton Quays, Peterborough, PE2 8YY UK

**Keywords:** Habitat, Citizen Science, Biodiversity recording, Earth Observation

## Abstract

**Context:**

Reversing the global biodiversity crisis requires not only conservation and management of species, but the habitats in which they live. While there is a long history of biodiversity recording by volunteers, at least in Europe, information on habitats is less frequently recorded. Habitat data is needed to map and monitor habitat extent and condition; to train and validate earth observation (EO) data; and to explain biodiversity change. The complexity of habitat classifications means that it is challenging to record habitat well, but citizen science provides diverse opportunities to improve the range and scale of habitat recording.

**Objectives/Methods:**

We reviewed how citizen science can complement surveys by professionals and EO for habitat recording, and discuss its opportunities and challenges. We summarised a survey of 458 volunteer biodiversity recorders asked about their interest in and barriers to recording habitat. From this we developed a framework of questions to design citizen science that is appropriate and effective for habitat recording.

**Results/Conclusions:**

We found that existing biodiversity recorders were willing to consider habitat recording, but many lacked confidence and some lacked motivation. Our framework of six questions addresses the interplay between volunteer motivation and confidence, and data accuracy. It highlighted design considerations such as clarity of purpose, cost to volunteers, protocol complexity and scale of sampling. This impacts the training and support required by volunteers. Building this understanding into citizen science design enables us to develop activities that meet the needs for habitat data for monitoring, EO validation and research.

**Supplementary Information:**

The online version contains supplementary material available at 10.1007/s10980-025-02155-4.

## Introduction: why record habitat?

In the face of global declines in nature, monitoring is a crucial activity to support environmental decision-making (Butchart et al. 2010; Johnson et al. 2017). Habitat can be defined as “the place in which an organism lives, which is characterised by its physical features or by the dominant plant types” (Martin and Hine 2008). Habitats are a foundational component of ecosystems, so habitat alteration (changing habitat type and condition) is an important driver of biodiversity change (Reid et al. 2005, Burns et al. 2023, Shaw et al. 2024), especially for habitat specialists (e.g. Clavel et al. 2011). This means that monitoring change in habitat coverage, extent and condition is important (Bunce et al. 2013, Newbold et al. 2015, 2019) but habitat monitoring receives much less focus than species monitoring (Moersberger et al. 2022). Collecting habitat data in the field can be complex and expensive (Lengyel et al 2008), and even the expansion of remote sensing cannot currently answer all the questions of interest (Pettorelli et al. 2014; Stephenson 2020). Citizen science, i.e. the involvement of volunteers in collecting data, is increasingly recognised as a cost-effective tool to support environmental science through large-scale data collection (Roy et al. 2014, Theobald et al. 2015). It is clear that citizen science is a somewhat untapped resource to meet the needs of habitat mapping and monitoring, both by enlisting existing biodiversity recorders to record habitat or recruiting volunteers to projects focused on habitat recording.

**Fig. 1:**
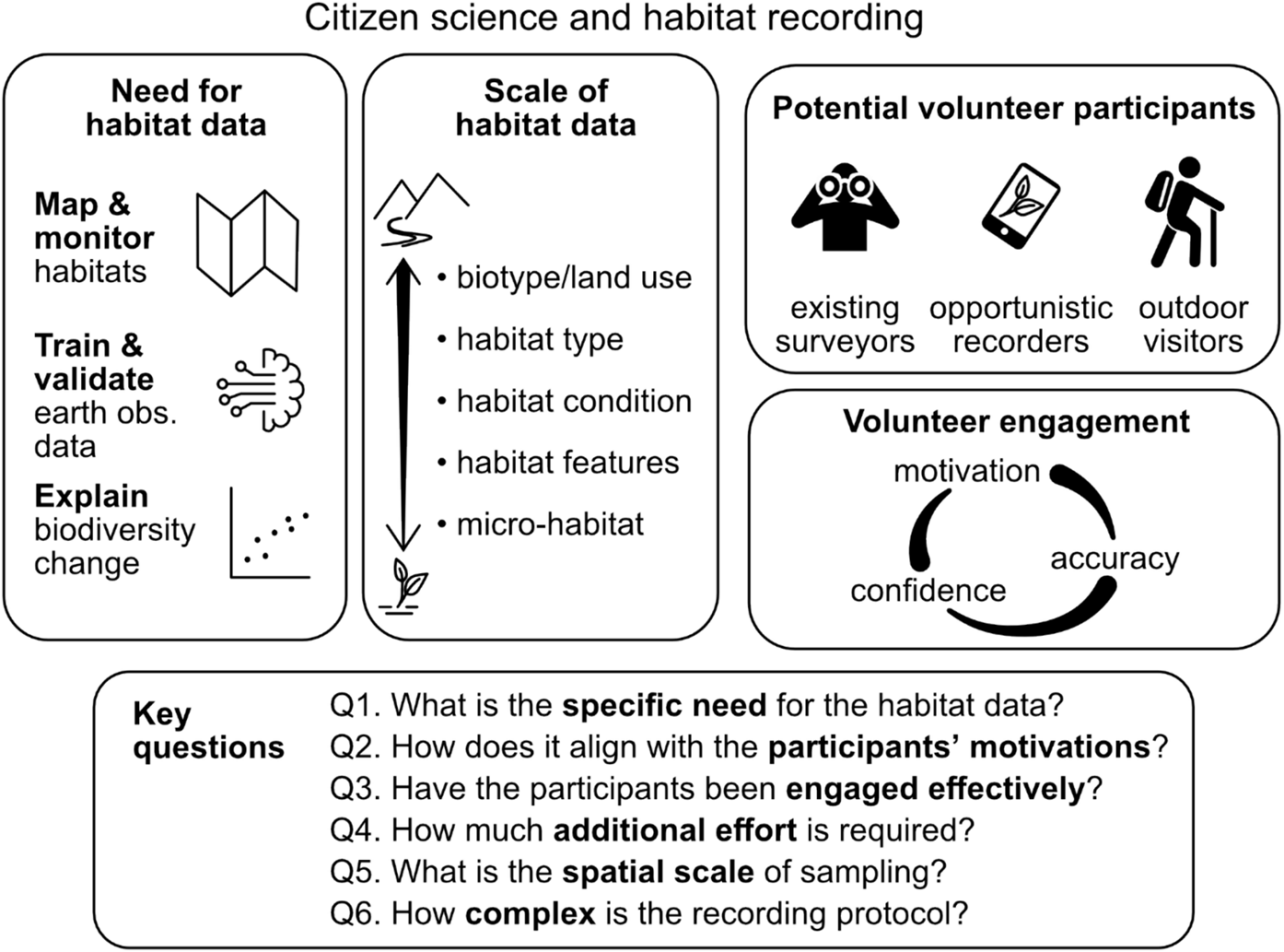
Overview of the paper, showing how the need for data (Sect. "Introduction: why record habitat?" in the text), scale of data (Sect. "A review of approaches for monitoring habitats"), and aspects of volunteer engagement (Sects. "Opportunities and challenges with engaging volunteers to record habitat in the field" and "A framework for considering citizen science for habitat recording"), support our key questions (framework; Sect. "A framework for considering citizen science for habitat recording") for considering citizen science for habitat recording. Motivation, accuracy and confidence is likely to differ between existing volunteer surveyors (i.e. those doing structured surveys of birds, plants etc.), opportunistic recorders (i.e. those recording as and when they choose), and outdoor visitors, such as hillwalkers or ramblers, who may not already formally record observations

Broadly, there are three main reasons to collect habitat data (Fig. 1). The first reason is to directly assess the extent, and condition of habitats, and their change over time. Although habitat extent is well-documented in places like Europe (Mücher et al. 2009) this is not true everywhere: detailed knowledge on habitat extent is patchier in other parts of the world (Pocock et al. 2018; Jung et al. 2020). Sometimes monitoring habitat change is even a legislative requirement, e.g. monitoring of “priority habitats” mandated under the European Union Habitats Directive (92/43/EEC) (Ellwanger et al. 2018). Many current conservation efforts focus on habitats—from restoring degraded habitat to creating new habitats through targeted management action, such as ‘biodiversity net gain’ (zu Ermgassen et al. 2021) or ‘rewilding’ (Carver et al. 2021)—so direct monitoring of habitat change is beneficial. Additionally the emphasis, at least in the UK, to ‘natural capital assessment’ is often underpinned by a habitat-based, rather than a species-based, assessment (e.g. Curnow 2019).

The second reason for collecting habitat data is that they are needed to validate other sources of landscape monitoring data (typically from remote sensing, e.g. Bell et al. 2015, but see Foody 2024 for discussion about ground-truthing). Given the challenges of monitoring habitat in the field, there has been rapid growth in the use of remote-sensed data from Earth Observation (EO; Corbane et al. 2015; Hassall et al. 2022). These data have increased dramatically in quality and frequency of updates over the past decade as the technology of satellites and unmanned aerial vehicles (‘UAVs’/drones) has advanced (Kuenzer et al. 2014; Wulder et al. 2018). EO scientists require field-based data to train the classification algorithms and validate outputs before upscaling predictions to create comprehensive habitat maps (Leibovici et al. 2017; Enterkine et al. 2022). Furthermore, many measures of habitat condition require on-the-ground observations to supplement EO data on habitat extent (Gerard et al. 2015), although see van der Plas et al. (2025) on how species data can be combined with EO to upscale habitat assessment.

Third, habitat data are needed to understand habitat associations with individual species, to assess the role of habitat change as a driver of biodiversity loss (Burns et al. 2023) and develop plans to reverse these declines (Leclère et al. 2020). There is a long history of natural historians recording detailed habitat attributes, co-located with species records, to gain a detailed understanding of species ecology (Webb and Lott 2006; Veech 2021; Ashwood et al. 2024) which supports this aim.

Here we consider the potential of citizen science for habitat recording. First, we consider the challenges and opportunities for habitat monitoring. Second, we report on a survey of 458 current and potential biodiversity citizen scientists in the UK to understand their motivations and confidence relating to recording habitat. We focus on the UK as a case study, because it already has a high level of interest in citizen science and biodiversity monitoring, and so there is potential to gain added benefit from the volunteers already doing biodiversity recording in the field. Third, we present a framework (Fig. 1) to test when citizen science will be suitable for habitat monitoring to help guide those considering developing new projects.

## A review of approaches for monitoring habitats

### What do we mean by habitat?

Despite ‘habitat’ being an intuitively simple concept, there is vast complexity in what people actually mean when they refer to ‘habitat’ (Table 1). The term is commonly used to refer to “habitat type”, which (in terrestrial systems) is typically defined by the combination of bio-physical features, vegetation type and structure, and the form of human management (Krausman and Morrison 2016). In practice, definitions of habitat (in the broadest sense) are scale-dependent, ranging from large-scale biotypes across landscapes, to small-scale details of the bio-physical environment and ‘microhabitat’, typically with reference to individual species’ needs (Fig. 1; Hall et al. 1997; Diaz et al. 2004).

**Table 1:** Characterisation of how ‘habitat’ can be described in different ways, and suitability of volunteer and earth observation (EO) recording

Aspect of habitat	Description	Feasibility for field surveyors (contracted staff or volunteers)	Feasibility for EO
Physical Habitat	Associated with the geographical location, such as elevation, aspect, soil type, drainage etc., especially relevant for plants, some invertebrates, fish	Generally, not efficient use of time for field surveyors in regions such as Europe because good data are already available from existing surveys and EO	Mostly excellent. Increased availability of high-resolution satellite data and field surveys for training and validation datasets makes this more possible at increasingly fine scales
Habitat Type	Mostly vegetation type/cover, either natural or anthropogenic, more relevant for some invertebrates and many vertebrates. At broad extents this is called the biotype	Feasible at a broad level, although can be tricky for ecotones and successional habitats for **untrained volunteers**. Alternatively, via collecting more detailed plant data (e.g. species abundance in quadrats)	Resolution varies according to habitat, often good for many broad-scale habitats, but scarce habitats (e.g. reedbed) can be difficult to accurately identify
Land use/land cover	Often synonymous with habitat type, although more commonly related to distinguishing human-dominated land use, including urban and different agricultural land uses	Feasible at a broad level, although can be difficult if seasonal to differentiate e.g. crop type	Same as above
Habitat Structure	Either characteristics (e.g. % vertical stems) or complexity, and partly independent of vegetation type; likely to influence community composition and abundance (rather than just presence) of many species	Task typically undertaken by trained, **contracted surveyors.** May be too time-consuming to record in detail for **volunteers** but could be recorded if simplified features can be defined	Often low, but can be good for some aspects e.g. woodland type or vegetation complexity/structure (e.g. LiDAR)
Habitat Features	Presence of specific elements in the habitat e.g. presence of dead trees in woodland or anthills in grassland, or amount of bare ground	Good where features are discrete things (e.g. number of standing dead trees) or recorded as presence/ absence (e.g. ash trees with dieback), harder where the scale is continuous (e.g. area of coverage) due to inter-personal variation	Depends on resolution of recording, better for continuous scale of measurement
Habitat Condition	State of the habitat, reflecting its management, and its ability to support valued elements of biodiversity. It may include elements of the components listed above (e.g. sward structure in grassland) and could include presence of positive or negative indicators species, such as the presence of invasive species, or indictors of other pressures	Good if recorded indirectly through presence of indicator species (plants or animals), less good for quantifying underlying drivers (e.g. level of fertilisation)	Generally poor

Assessing habitat ‘condition’ (e.g. based on habitat features) or ‘quality’ (e.g. with respect to suitability for focal species) is also valuable. This relies upon a judgement to define what ‘good’ is with regards to habitat features or associated species (Table 1). Because many definitions of habitat type are related to plant community composition, plant monitoring provides a valuable way to assess change in habitat type and condition/quality, such as already undertaken by volunteers in the UK’s National Plant Monitoring Scheme (NPMS; Pescott et al. 2015, 2019).

It is challenging to define (and record) habitat types accurately and consistently (Lengyel et al. 2008), partly because ‘habitat’ is, to some degree, a human-imposed definition upon the rich continuum of ecological complexity (Table 1). There have been many attempts to come up with common descriptors of habitat type (Table S1 for examples from the UK) but there is no ‘one-size-fits-all' approach. It is especially challenging where habitats are transitional or occur as small patches (e.g. Hou and Walz 2016). Many classification schemes are hierarchical and have high concordance at the general levels (e.g. woodland, grassland, etc.), but diverge at finer classifications, reflecting their different purposes (Table S1). When the purpose of the habitat monitoring is clearly articulated, appropriate habitat classifications can be selected. Using standardised classifications enhances the re-usability of the data for other purposes in the future.

### How do we record habitat?

Information on habitat extent, condition and habitat features can come from different sources. First we consider in-field surveys (focussing on professionals) and remote sensing, each of which have their benefits (Table 1), before considering citizen science in the following section.

Traditionally, habitat recording was often undertaken by contracted, professional surveyors (Ichter et al. 2014) or skilled volunteers who undertake landscape habitat mapping (which requires great experience and field skills), or plot-based monitoring, often of plant composition at set locations that are revisited across time. Examples include the LUCAS network across Europe (Pflugmacher et al. 2019) or the Nationwide Forest Inventory in the USA (Hoover et al. 2020). Relying on contracted surveyors is expensive and can only ever provide information on a subset of the area of interest.

More recently, remote sensing via earth observation (EO) has become a vital source of data, gathered by an increasing range of technologies, such as multispectral imaging (covering visual and non-visual spectra), or LiDAR (providing information on vegetation height or structure; Acebes et al. 2021), and with the sensors deployed on satellites, aircraft or UAVs (Hernandez-Santin et al. 2019). EO data is well-suited to describing land cover and land change at large scales (Wulder et al. 2018, 2020; Punalekar et al. 2024) and assessing some components of habitat composition or structure (Coops et al. 2020; Barnes 2021). EO data is typically available with complete spatial coverage in a gridded format that can be applied consistently across sites, at high spatial resolution, large extent and high temporal frequency (Kimm et al. 2020; Merrington et al. 2021).

Despite the advantages of EO data, in-field habitat recording is still required, especially to obtain datasets to train classification algorithms and to validate their results (Morton et al. 2011), and there is growing interest in citizen science to meet this need. Machine learning and improved image quality have great potential in making even more use of EO data (van der Plas et al. 2025), but some conservation-relevant habitat features are difficult (or currently impossible) to assess with EO data and habitats that are fragmented or have non-distinctive spectral reflectivity can present challenges for EO (Davies et al. 2024). Further use of EO data simply increases the need for in-field recording of habitat for training and validation data (Punalekar et al. 2024).

## Opportunities and challenges with engaging volunteers to record habitat in the field

###  Using citizen science for habitat recording

A huge number of volunteers already participate in environmental and biodiversity citizen science. It is often more cost-effective than contracting professional surveyors (Roy et al. 2014) and provides added benefits such as participant well-being and engagement with environmental change (Greenwood 2007; Pocock et al. 2023). These projects include both structured monitoring (following a protocol and revisiting defined locations) and unstructured recording (recording what you like, when you like; Pocock et al. 2017).

Reference to inventories such as Scistarter (https://scistarter.org/) and EU Citizen Science (https://eu-citizen.science/) shows that most environmental citizen science projects are focussed on recording species. However, there is a smaller, but diverse, range of projects that involve people in recording habitat. For instance, recording habitat condition of estuaries (Wharton et al. 2023), habitat features in protected areas (Kallimanis et al. 2017), mapping mosquito breeding habitats (Low et al. 2021), and many projects assessing river water quality (e.g. Joaquin 2018, Bishop et al. 2025). In the UK, some monitoring schemes already engage volunteers in habitat recording, for example, monitoring plant communities by annually recording in quadrats (Pescott et al. 2019) or recording habitat type and management along breeding bird transects (Martay et al. 2018). Studies show that volunteers can provide accurate data when following detailed protocols for habitat assessment (Kallimanis et al. 2017; Martay et al. 2018). Some successful projects in the UK go further and provide focused training for volunteers to go and map habitat (Habimap; https://www.gloucestershirewildlifetrust.co.uk/habimap) or collect quadrat data to train EO (Space4Nature; https://www.surreywildlifetrust.org/space4nature). Meanwhile fixed-point repeat photography can engage a much wider range of non-experts (Flowers et al. 2023; Harley & Kinsela 2022). The collection of ground-based imagery has potential for wider crowd-sourcing of data to validate EO, but is largely untested (Morueta-Holme et al. 2024). Many thousands of people annually take part in species recording (Di Cecco et al. 2021) and many others visit the countryside for work or leisure. If these people undertook relatively small tasks to record habitat, they could generate huge amounts of valuable data at great spatial coverage that would complement existing habitat-focussed projects.

Altogether then, citizen science could be an ‘untapped resource’ for habitat recording, but are people motivated to record habitat? In the UK, the BTO/RSPB/JNCC Breeding Bird Survey invited people to record habitat along the survey transects from its inception in 1995. While many recorders did do this, an increasing sizeable minority opted not to record habitat until 2014 when it became mandatory as part of online submission (Fig. S1). Despite this, feedback from organisers of several UK-based biodiversity surveys indicates that habitat recording is often not a popular task among many volunteers, hence our motivation to construct a framework to consider when and how to design effective citizen science for habitat recording.

### Survey of interest in habitat recording by volunteer biodiversity recorders

To discover more about volunteers’ attitudes to recording habitat, we undertook a survey of current and potential species recording volunteers in the UK. The survey was circulated via social media channels and mailing lists of JNCC and partner organisations. Over a three-month period in summer 2023, we received 458 responses to the survey (full details in Harris et al. 2024). 410 (90%) had participated in some form of biodiversity recording within the past year; of these, 55 (12% of the total) had participated in only structured recording schemes, 114 (25%) in only unstructured species recording, and 241 (53%) had participated in both. We gained responses from recorders of many taxonomic groups, but accept there was likely participation bias, with responses more likely from those more favourably disposed towards habitat recording.

**Fig. 2:**
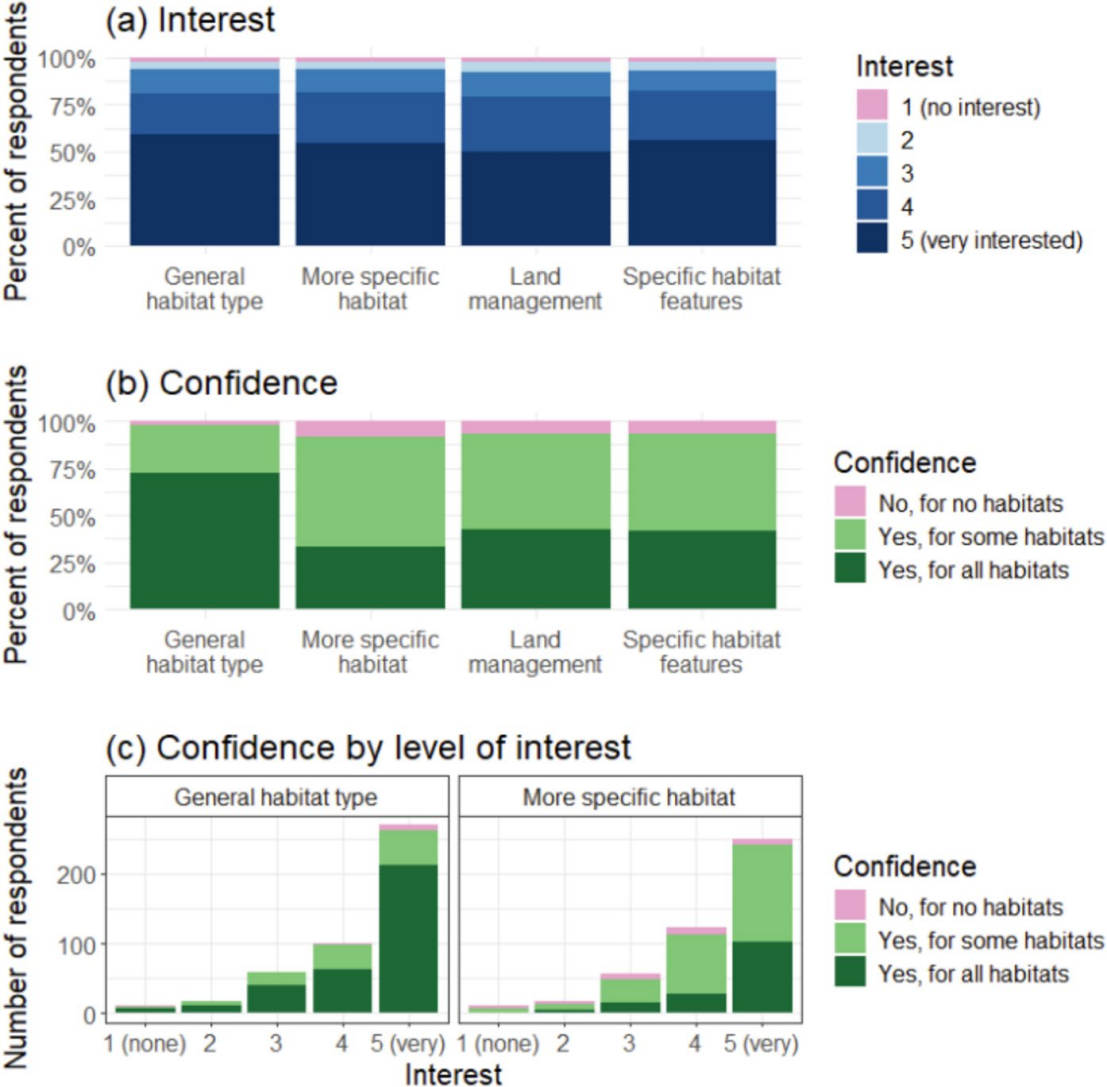
Survey responses to the question on a) interest in collecting habitat data (1 is no interest at all and 5 is very interested) and b) confidence of the respondents ability to record the following types of habitat data: general habitat (e.g. woodland or grassland), specific habitat type (e.g. oak woodland, calcareous grassland), visible habitat management (e.g. grazed or ungrazed) and specific habitat features (e.g. presence of dead wood, evidence of pollution). Number of respondents = 458 (redrawn from Harris et al. 2024)

We found that > 79% of respondents said that they were interested in recording habitat (scoring 4 or 5 on the five-point scale from 1 being ‘no interest at all’ to 5 ‘very interested’). Compared to the proportion of respondents who were ‘very interested’ in recording general habitat type (e.g. ‘woodland’; 59%), slightly fewer were ‘very interested’ in recording more specific habitat type (e.g. ‘oak woodland’) or specific habitat features (54% and 56%, respectively), and slightly fewer again were very interested in recording visible habitat management (e.g. ‘with livestock grazed’; 50%; Fig. 2a). Those who had not undertaken species recording in the past year stated that they were less interested in recording habitat (67% scored 4 or 5 for interest in recording ‘general habitat type’) than those who had done recording (84%; Harris et al. 2024).

We asked how confident the respondents felt in recording habitat type; the majority were confident in recording general habitat type for all habitats (74%), but when considering specific habitat type, visible land management and specific habitat features, most respondents (51–59%) were only confident “for some habitats” (Fig. 2b). In general, those who had lower confidence also had lower interest in recording habitats (Kendall’s tau = 0.142 and 0.224 for general and more specific habitat, respectively, both with *p* < 0.001).

When asked about the barriers to collecting habitat data, the top two responses were lack of time in the field (48%) and lack of confidence in identifying/assessing habitats (41%; Fig. S2a). In order to boost their confidence in recording habitats most respondents (78%) indicated that they would like instructions to follow in the field, while others wanted ongoing support (57%) and recorded online training (41%); fewer people (< 30%) selected interactive online training, in-person training in the field or online quizzes (Fig. S2). Crucially, when told that habitat recording would take no longer than five minutes (i.e. general recording of broad habitat type or simple-to-record habitat features, rather than quantitative assessment of vegetation cover or other habitat features), the majority of respondents stated that they preferred to record habitat at the same time as current species recording (70%) or as part of their existing visits (before or after recording; 31%), while few (< 18%) would be happy to do this during a new or an additional visit (Fig. S2c).

In summary, the survey indicated that there is potential to expand the extent to which biodiversity recorders to do habitat recording, but this finding will need to be tested through pilot projects to ensure recording of target taxa is not compromised. Putting this into practice requires greater consideration of the nature of the habitat recording and the motivation of the potential volunteers. Therefore, we propose a framework of six guiding questions, based on our experience, that organisers can use to assess the use of citizen science for habitat recording.

## A framework for considering citizen science for habitat recording

The rapid growth in participation in biodiversity citizen science indicates that there is potential for citizen science to contribute to habitat recording for different purposes and there appears to be a willingness of volunteers to record habitat. However, as we have discussed, recording habitat is not straightforward, and evidence from scheme organisers suggests that existing biodiversity volunteers do so somewhat reluctantly (Fig. 2), although clear instructions and supporting material could enhance both interest and confidence. This means that, when developing citizen science for habitat recording, we should design so that participants’ motivations and the researcher-oriented goals can align (Domroese and Johnson 2017; Johnson et al. 2018). This needs to consider the inter-play between the usefulness of the data (including its accuracy), the complexity of the protocol, and the confidence and commitment of the volunteer which will entail appropriate support and training. Therefore, drawing inspiration from previous guidance on ‘using citizen science’ (Pocock et al. 2014), we have constructed a framework of six key questions to help guide those considering using citizen science for habitat recording (presented in Fig. 1 and expanded in Box 1).

Fundamental to any form of monitoring is having a clear aim for what is being recorded and the purposes for which it will be used (Lindenmayer et al. 2012); for example, whether to map habitat types, monitor habitat condition and/or change, or validate EO data (Fig. 1; Question 1 in Box 1). Each has different data needs: e.g. in spatial coverage, co-location with biodiversity monitoring data, sample size, or detail of habitat classifications. Being precise about the data requirements helps organisers carefully consider the suitability of citizen science for the task, but they should explicitly consider the potential for re-use of data, e.g. aligning recording with existing habitat classifications, such as UKHab (UKHAB 2023), designed in the UK to integrate with all major European habitat classifications.

Volunteers in citizen science are contributing to activities with little or no direct compensation, beyond gaining personal satisfaction, e.g. though contributing to science or increasing knowledge (Peter et al. 2019; Thompson et al. 2023). Habitat recording represents a cost for volunteers and needs to be aligned with their motivations and supported by effective engagement (Questions 2 and 3; Tiago et al 2017; Maund et al. 2020). Broadly, there are three potential sources of participants for citizen science habitat recording (Fig. 1): i) existing surveyors who are already committed to record habitat or habitat features, or biodiversity volunteers who could record habitat as part of their survey (e.g. BBS; Martay et al. 2018, NPMS; Pescott et al. 2019); ii) opportunistic wildlife recorders who could also record habitat when out recording species, e.g. if habitat recording is built into smartphone applications; and iii) new audiences, e.g. outdoor visitors to places from which habitat data would be valuable (e.g. Bishop et al. 2025). This latter group might be more motivated by a sense of place, and environmental change in general (Newson et al. 2016), so requiring different ways of engagement than wildlife recorders. We expect that existing species recorders might be more strongly and consistently motivated to record habitat features associated with their species of interest, e.g. pond characteristics when recording dragonflies (Odonata). If the connection is less strong, e.g. being asked to record broad habitat type when undertaking focused insect surveys, then motivation may be lower, and organisers will need to carefully consider incentives for people’s continued engagement and retention in the project. Rates of change should also be considered: researchers might desire repeated, annual habitat recording, but if the habitat changes only slowly then then volunteers might lack the motivation to record the same information year after year.

Involving potential participants and data users as stakeholders in co-design from the earliest stage of a project is essential (Question 3; Skarlatidou et al. 2019). Fit-for-purpose training, engagement and support would increase people’s willingness to participate and would enhance data quality, while also increasing the participant’s knowledge and engagement with the underlying issues (Evans et al. 2005). For detailed monitoring, training could be provided in person to boost volunteers’ accuracy and confidence.

It is important to consider the level of additional effort required (Question 4) alongside other activities that the volunteer will be doing. For instance, a wildlife recorder might be happier to undertake detailed habitat assessment compared to a recreational hiker, as long as it does not distract from their primary motivation for being in the field, e.g. surveying their target taxa. In contrast, a recreational hiker might be willing to participate in crowd-sourcing of ground-based images, as long as the request is clear and not onerous (Morueta-Holme et al. 2024). In each case they would require targeted promotion to recruit sufficient recorders in the places where data are needed.

Any habitat recording needs to balance the subtlety and complexity of what might be seen in the field, with the need to classify simply and accurately in a way that meets the goals of the project organisers and potential future re-users of data (Question 5). As we have discussed, habitat classification is not straightforward (Table S1). Indeed, the UK’s NPMS provides volunteers with a detailed 64-page guidance on habitat identification (NPMS 2019), while the UKHAB classification recognises 119 habitat types (with 268 secondary codes) and runs to 545 pages (UKHAB 2023). Although respondents in our survey felt confident in recording broad habitats, they were less confident in recording detailed habitat types (Fig. 3b), furthermore, this was a self-assessment of confidence which may not translate into accuracy of recording (See et al. 2013; Kallimanis et al. 2017). Making classifications too complex may negatively impact volunteers’ confidence and their accuracy (important for validating remote sensed data; Foody 2024); and confidence is likely to be linked to motivation and long-term retention in projects. However, making it too simple may not be fulfilling for volunteers and can mis-represent if there are (i) multiple habitats within a location; (ii) habitats that intergrade or form mosaics (which will itself be scale-dependent: e.g. “woodland clearings” could be classified as “woodland” or “grassland” depending on the scale at which it is considered, Question 6); or (iii) habitats that are intrinsically hard to categorise.

By reflecting on the questions in our framework, project organisers can design activities that add value to our knowledge on habitats and meet specific needs for data and that are aligned with their participants’ motivations. For instance, when recording habitat to evaluate drivers of biodiversity change, it may be most important to focus on assessing specific features that cannot be effectively assessed with EO data, thus complementing (rather than replicating) this source of information; or, when collecting data to validate EO classifications, it is important to be specific about the need for the data (e.g. number of samples, from which locations) so as to optimise, and not waste, participants’ recording effort.

**Box 1** A framework for designing citizen science that is appropriate and effective for habitat recording **Q1. What is the specific need for the habitat data?**

- Is it targeted at specific (set of) species or environmental driver(s)? Is there a testable hypothesis?
- Has the spatial and temporal coverage and sample size been defined?
- Can the data requirements be reasonably met by volunteers, in terms of sample size, spatial coverage, accuracy and/or level of detail?
- Is it possible to use an existing habitat classification or standard measures of habitat features to support re-use of the data?

**Q2. How does it align with the participant’s motivations?**

- Have specific potential audiences been defined? What are their motivations for participation?
- Have potential volunteers been consulted, or their experiences referred to, in devising the recording the system?
- Has the proposed recording activity been tested with potential volunteers to ensure that they are motivated and can collect the data confidently and accurately?
- If there is a prescribed frequency for re-recording, does it match with volunteers' willingness to record?
- Is training provided to support volunteers in collecting data of known accuracy?

**Q3. Have the participants been engaged effectively?**

- Is the need for the data clearly communicated to the potential volunteers?
- Does the activity align with the reasons that people are in the field, e.g. for birdwatchers, using the data to inform bird conservation, and is this communicated effectively?
- Has the retention of participants in the activity been considered, for example providing feedback about the use of the data?

**Q4. How much additional effort is required?**

- Does the timing align with existing visits, or are separate visits required?
- Can it be done alongside existing recording, or will it distract attention from the main purpose?
- Can it be done without additional equipment (including e.g. an identification key/guide)?
- Does it conflict with the participant’s existing activity, e.g. birdwatching or hiking?

**Q5. How complex is the protocol?**

- Is the habitat or feature readily recognisable, or does it require expertise/background knowledge?
- Do simple categories match what observer sees, or are qualitative judgements required?
- Can recorders be confident in their assessment, and is their assessment accurate?

**Q6. What is the scale of sampling?**

- Is the sampling based on features at a single point, or over a larger area?
- How well-defined and identifiable is the sampling point/area ‘in the field’?
- Does recording occur in a single habitat block, or amidst a mosaic/transitional landscape?
- Is annual/regular sampling required and is the seasonal timing appropriate?

## Conclusion and future opportunities

New technology is transforming what is possible for citizen science and its data (Lahoz-Monfort and Magrath 2021, Sheard 2024, van der Plas et al. 2025). This means that, for example, digital photographs can be an effective way to crowd-source habitat recording (e.g. Chronolog 2024; Harley and Kinsela 2022; Morueta-Holme et al. 2024), with crowd-sourcing of image classification, possibly in combination with AI, offering the potential to efficiently utilise vast datasets (Lahoz-Monfort and Magrath 2021). Accurate measures of habitat structure could soon be obtained from citizen science through 3D photogrammetry, structure from motion or handheld LiDAR, such as that already integrated in premium smartphones (Jaud et al. 2019; Enterkine et al. 2022; Tavani et al. 2022). Technology can also help to target in-field sampling by citizen science. Records of habitats from some locations will be more valuable than others, e.g. visiting sites that have been visited in the past, or those with high classification uncertainty to improve the EO analytic algorithms. Adaptive sampling approaches can help to optimise citizen science sampling strategies (Mondain-Monval et al. 2024) and could be linked to dynamic tools on smartphones including augmented reality (overlaying information on targets to live images on smartphones). It would be valuable to explore how effective it would be to combine this with gamification or adaptations of geocaching, especially for new audiences (Dunlap et al. 2015; Laso Bayas et al 2016; Preece 2017).

Habitat is an important, multi-scale attribute of the natural world, although it can be hard to classify precisely. Citizen science is a natural approach to support more collection of habitat data for monitoring and research. However, there are many uses of the data and many potential audiences: the motivations for each audience with each application will differ, and so our framework (Sect. "A framework for considering citizen science for habitat recording") should help organisers be clear about their need for the data, how they engage with potential volunteers, and how they assess the effort required and the complexity of the protocol. All of this affects the training and support that volunteers will require, and hence the accuracy of data and volunteers’ confidence, and ultimately their motivation. Building these understandings into our design of citizen science habitat recording will enable us to propose and test better activities to meet the needs for habitat data to support monitoring and research.

## Supplementary Information

Below is the link to the electronic supplementary material.

Supplementary file1 (DOCX 264 KB)

## Data Availability

The survey data reanalysed in this report were published as part of the JNCC report Harris et al. (2024) and can be found here: https://hub.jncc.gov.uk/assets/66f5a314-b5ee-462b-ae16-22e527d3b14c.

## Declarations

### Statements and Declarations

Ethical approval for the survey was gained from the UK Centre for Ecology and Hydrology Ethics Committee prior to circulation.
