# Supplementary material for: Not just species recording: the potential of citizen science for habitat monitoring
Source: Landsc Ecol. 2025 Aug 26;40(9):181. doi: 10.1007/s10980-025-02155-4 (PMC12380648; doi:10.1007/s10980-025-02155-4)

# Supplementary Material

Table S1. Two example habitats from the UK and how they could be classified according to different habitat classification schemes to demonstrate the complexity of classifying habitat type. Many of these are strictly hierarchical (e.g. EUNIS and CORINE), although not all subdivisions are nested (e.g. BBS; Breeding Bird Survey).

| Habitat Example | BBS^1^ | NPMS^2^ | JNCC Phase 1^3^ | NVC^4^ | EUNIS^5^ | CLC^6^ | UKHab^7^ |
| --- | --- | --- | --- | --- | --- | --- | --- |
| Oak woodland, with understorey | A. Woodland;  1. Broadleaved;  1. Mixed-aged or semi-natural &  8. Low disturbance;  1. Dense shrub layer & 9. Dead wood present | 1. Broadleaved woodland;  Dry deciduous woodland;  canopy species Oak, shrub species = blackthorn, hawthorn, hazel | A. Woodland and scrub  1. Woodland  1. Broadleaved  1. Semi-natural | W10 –Quercus robur-Pteridium aquilinum-Rubus fruticosus woodland | G. Woodland, forest and other wooded land;  G1. Broadleaved deciduous woodland;  G1.A1 Oak-ash-hornbeam woodland on eutrophic and mesotrophic soils;  G1.A11 Mixed Atlantic bluebell oak forests | 3. Forests and semi-natural areas;  3.1 Forests,  3.1.1 Broadleaved Forest | W Woodland and forest;  w1 broadleaved mixed and yew woodland;  w1f Lowland mixed deciduous woodland;  w1f5 Dry oak-dominated woodland |
| Grazed pasture, with no hedges | E. Farmland;  1. Apparently improved grassland;  4. Other field boundary (wall, ditch, etc.);  3. Sheep | 4. Lowland Grassland;  Neutral pastures and meadows;  list species present grasses (e.g. Crested Dog’s-tail) and herbs e.g. buttercups, Oxeye daisy, red clover | B. Grassland and marsh  2. Neutral Grassland  2. Semi-improved | MG6 – Lolium perenne Cynosurus cristatus grassland | E. Grasslands and lands dominated by forbs, mosses or lichens;  E2. Mesic grasslands;  E2.1 Permanent mesotrophic  pastures and aftermath grazed meadows;  E2.12 Ditch-broken pastures | 2. Agricultural areas:  2.3 Pastures;  2.3.1 Pastures | G Grassland;  g3 Neutral grassland; g3a Lowland meadows  or  C Cropland;  c1b temporary grass and clover leys |

^1^ Breeding Bird Survey - Crick (1992; BTO 2022); ^2^ National Plant Monitoring Scheme - https://www.npms.org.uk/sites/default/files/PDF/NPMS_Survey%20Guidance%20notes_WEB_2ndEd.pdf; ^3^ Joint Nature Conservation Committee, Phase 1 habitat survey - https://data.jncc.gov.uk/data/9578d07b-e018-4c66-9c1b-47110f14df2a/Handbook-Phase1-HabitatSurvey-Revised-2016.pdf; ^4^ National Vegetation Classification – Rodwell (2018); ^5^ European Nature Information System – Davies et al. (2008) https://eunis.eea.europa.eu/; ^6^ CORINE Land Cover - https://land.copernicus.eu/pan-european/corine-land-cover; ^7^ UK Habitat Classification System – <https://ukhab.org>

**Figure S1** Number of sites surveyed for the Breeding Bird Survey (BBS) for which habitat data were submitted in each year since the scheme’s inception in 1994. (Note, public movement restrictions reduced coverage in 2001 (foot and mouth disease) and 2020 (COVID-19)). Habitat recording was made mandatory for online recording in 2014 (red dashed line).


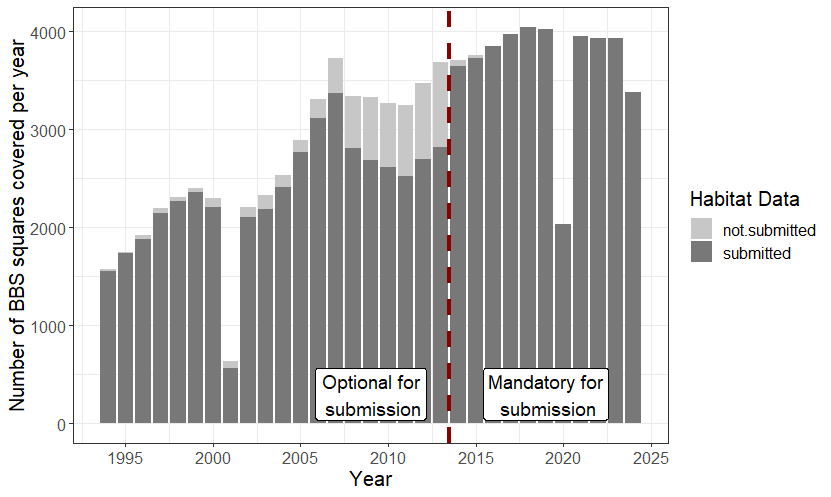


**Figure S2** The number of responses for a) barriers to collecting habitat data, (b) preferred method to receive training and (c) preferred time to collect habitat data. Each respondent could select multiple options (up to three for part a b; see Harris et al. 2024 for survey questions).


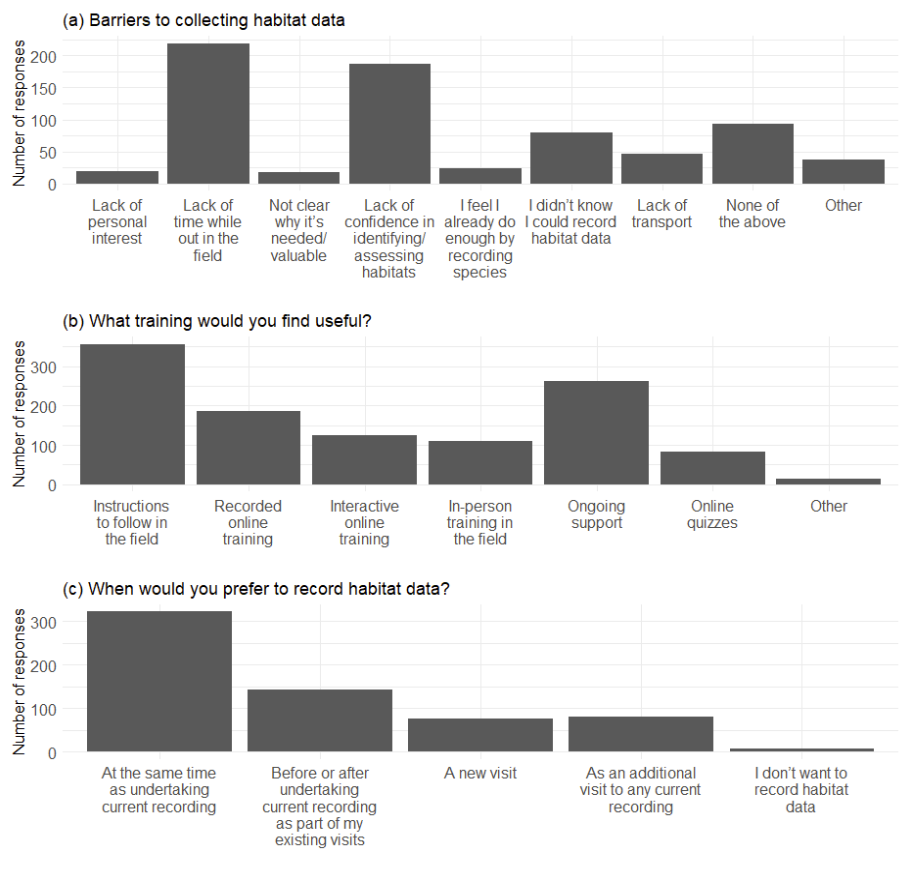

Supplement: Supplementary file 1 — Supplementary file1 (DOCX 264 KB) [file 10980_2025_2155_MOESM1_ESM.docx]
